# The Co-Evolution of Sleep and Diet: Toward an Emerging Framework of Evolutionary Chrononutrition in Circadian–Metabolic Health

**DOI:** 10.3390/nu18121947

**Published:** 2026-06-16

**Authors:** Nicola Luigi Bragazzi, Halil İbrahim Ceylan, Alice Rosi, Francesca Scazzina, Andrea de Giorgio, Ismail Dergaa, Egeria Scoditti, Sergio Garbarino

**Affiliations:** 1 Human Nutrition Unit (HNU), Department of Food and Drugs, University of Parma, 43121 Parma, Italy; alice.rosi@unipr.it (A.R.); francesca.scazzina@unipr.it (F.S.); 2 Physical Education and Sports Teaching Department, Faculty of Sports Sciences, Atatürk University, 25240 Erzurum, Türkiye; halil.ibrahimceylan60@gmail.com; 3 Artificial Engineering, 80121 Naples, Italy; andrea@degiorgio.info; 4 Higher Institute of Sport and Physical Education of Ksar Saïd, University of Manouba, Manouba 2010, Tunisia; phd.dergaa@gmail.com; 5 Physical Activity Research Unit, Sport and Health (UR18JS01), National Observatory of Sports, Tunis 1003, Tunisia; 6 Institute of Clinical Physiology (IFC), National Research Council (CNR), 56124 Pisa, Italy; egeria.scoditti@cnr.it; 7 Department of Neuroscience, Rehabilitation, Ophthalmology, Genetics and Maternal/Child Sciences (DINOGMI), University of Genoa, 16132 Genoa, Italy; sgarbarino.neuro@gmail.com

**Keywords:** sleep, diet, chronobiology, circadian rhythms, evolutionary medicine, evolutionary chrononutrition

## Abstract

Sleep and dietary behavior are deeply conserved biological processes that co-evolved under ecological pressures shaping human anatomy, metabolism, immunity, cognition, and life history strategies. Major transitions in human dietary ecology, including plant-dominant hominin foraging, increased meat consumption, control of fire and cooking, agricultural domestication, industrialization, and postindustrial globalization, restructured nutrient intake, pathogen exposure, microbial ecology, metabolic demands, and temporal organization of behavior. Emerging evidence from evolutionary genomics, chronobiology, neuroendocrinology, and microbiome science indicates that sleep–feeding interactions represent a conserved adaptive regulatory module optimized for fluctuating energy availability and strong photoperiodic entrainment. Modern environments characterized by widespread availability of highly palatable, energy-dense foods rich in refined carbohydrates, added sugars, and multiple industrial additives, together with artificial light at night, continuous caloric access, sedentary behavior, and psychosocial stress produce a profound evolutionary mismatch destabilizing circadian–metabolic homeostasis. This mismatch is characterized by circadian disruption, temporal misalignment of feeding and sleep behaviors, and, in many populations, insufficient sleep duration. Within this conceptual landscape, the emerging framework of “evolutionary chrononutrition” proposes that metabolic health and sleep integrity depend not only on what humans eat, but critically on when food is consumed in relation to endogenous circadian architecture shaped across deep evolutionary time. This review synthesizes anthropological, physiological, and molecular evidence to develop an integrative evolutionary framework linking sleep and diet to contemporary cardiometabolic, neurodegenerative, inflammatory, and psychiatric disorders, with particular emphasis on how each major dietary transition plausibly altered sleep duration, architecture, circadian timing, neuroendocrine regulation, and the temporal alignment between feeding behavior and biological rhythms.

## 1. Introduction: Sleep and Feeding as Co-Evolved Adaptive Systems

Sleep is a highly conserved biological state observed across a wide range of *taxa*, including mammals, birds, reptiles, fish, and even invertebrates, such as the arthropod *Drosophila melanogaster* and the nematode *Caenorhabditis elegans* [1]. This underscores its deep evolutionary roots and strong adaptive value despite the apparent costs associated with behavioral quiescence and increased vulnerability to predation [2,3]. Such remarkable phylogenetic persistence suggests that benefits conferred by sleep must outweigh its ecological risks, and multiple, non-mutually exclusive theoretical frameworks have been proposed to account for its evolutionary maintenance [4,5].

Among these, the “synaptic homeostasis hypothesis” [6,7] posits that wakefulness is associated with a progressive net potentiation of synaptic connections driven by learning and environmental interactions, whereas sleep serves to renormalize this synaptic strengthening, thereby preserving neuronal efficiency, signal-to-noise ratios, and overall network stability while facilitating memory consolidation.

In parallel, converging experimental evidence indicates that sleep enhances the activity of the glymphatic system [8,9], promoting the clearance of neurotoxic metabolites such as β-amyloid and other by-products of neuronal metabolism, which accumulate during wakefulness and may contribute to neurodegenerative processes if not efficiently removed [10,11]. Sleep has also been increasingly recognized as a critical regulator of immune function, modulating both innate and adaptive immune responses through effects on cytokine production, inflammatory signaling, and immunological memory, thus contributing to host defense and long-term health [12,13].

Importantly, sleep is tightly intertwined with energy balance and metabolic regulation [14,15,16]. Feeding behavior and sleep–wake regulation are orchestrated by partially overlapping neural circuits centered in the hypothalamus [17], where peripheral metabolic signals, including leptin, ghrelin, insulin, and glucose, are integrated with neuropeptidergic systems such as neuropeptide Y, melanocortin pathways, and orexin/hypocretin signaling [18]. These networks coordinate arousal, appetite, energy expenditure, and circadian rhythms, reflecting a deeply integrated evolutionary architecture linking sleep and nutrition [17,18]. Orexin-producing neurons, in particular, play a pivotal role in stabilizing wakefulness while simultaneously influencing food-seeking behavior, thereby acting as a functional bridge between arousal states and energy homeostasis [19,20,21].

Experimental sleep restriction paradigms in humans demonstrate that curtailed sleep leads to reductions in circulating leptin levels and elevations in ghrelin concentrations, hormonal changes that collectively enhance subjective hunger, increase appetite, particularly for energy-dense foods, and potentially promote positive energy balance [22]. Together, these findings highlight sleep not merely as a passive state of rest but as an active and metabolically integrated process central to neurobiological maintenance, immune competence, and the regulation of feeding behavior.

The co-localization and dynamic interaction of neural circuits governing sleep and feeding strongly imply that these systems did not evolve independently but rather emerged as functionally integrated adaptive modules. Hypothalamic and brainstem nuclei involved in arousal, satiety, and reward processing exhibit extensive anatomical overlap and reciprocal connectivity, enabling bidirectional modulation between metabolic state and vigilance [23,24]. Such integration allows organisms, including humans, to flexibly allocate behavioral time between foraging and rest in response to environmental constraints, energetic demands, and internal physiological signals.

From an evolutionary standpoint, this architecture would have conferred substantial fitness advantages by optimizing the temporal organization of feeding and recovery processes, ensuring that energy acquisition, tissue repair, immune defense, and neural recalibration occur in coordinated rather than competing phases. At the molecular level, circadian clocks provide the temporal scaffold upon which this integration is organized. Endogenous oscillators, operating through transcription–translation feedback loops, synchronize central and peripheral tissues to environmental light–dark cycles while simultaneously regulating metabolic gene expression, hormonal secretion, and substrate utilization [25]. Core clock genes modulate pathways involved in glucose homeostasis, lipid metabolism, mitochondrial function, and inflammatory responses, thereby linking temporal regulation to energetic efficiency [26,27].

In turn, nutrient availability and feeding timing feed back onto circadian machinery, influencing clock phase, amplitude, and peripheral synchronization [28]. This bidirectional coupling ensures that energy intake, digestion, nutrient partitioning, and sleep–wake cycles are aligned with predictable ecological and circadian rhythms, such as daylight availability and temperature fluctuations [28,29].

The evolutionary inseparability of dietary ecology and sleep physiology becomes particularly evident when considering species-specific adaptations. Nocturnal and diurnal organisms display distinct temporal feeding patterns tightly aligned with their circadian phase, and disruptions to this alignment, whether through artificial light exposure, shift work, or irregular eating schedules, lead to measurable metabolic dysregulation [30]. Such findings underscore that sleep timing, meal timing, and metabolic processing co-evolved under selective pressures favoring temporal coherence. The coordination of anabolic and catabolic processes with rest–activity cycles enhances energy efficiency, minimizes oxidative stress, and supports cellular repair mechanisms that are preferentially activated during sleep [31,32].

Taken together, the anatomical, cellular, molecular, biochemical/physiological, and ecological convergence of sleep and feeding systems supports the view that they represent a unified adaptive complex rather than discrete domains. Sleep cannot be fully understood without reference to metabolic context, just as dietary behavior cannot be disentangled from circadian and arousal dynamics. In evolutionary terms, the regulation of “when to eat” and “when to sleep” reflects a shared biological logic aimed at maximizing survival, reproductive success, and long-term organismal resilience.

**Figure 1:**
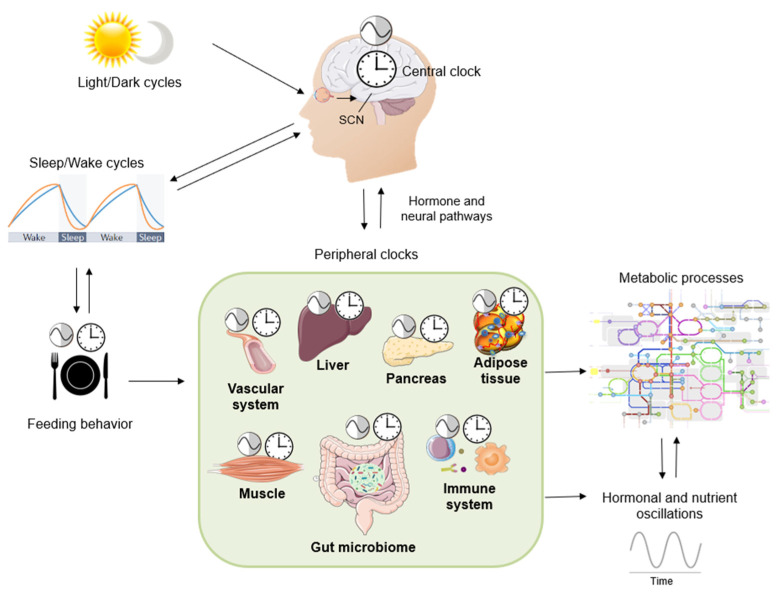
Integrated framework of sleep and metabolic health. Temporal alignment between environmental light/dark cycles, sleep/wake cycles, and feeding rhythms is organized through central and peripheral clocks to ensure an evolutionarily advantageous coordinated regulation of energy acquisition, hormonal changes, substrate utilization, tissue repair, immune defense, and neural circuits. The co-evolution and temporal organization of sleep and feeding patterns (“evolutionary chrononutrition”) optimize fitness and metabolic physiology. SCN: suprachiasmatic nucleus.

The present integrative review aims to provide the conceptual foundation for examining how modern alterations in diet composition, meal timing, and sleep patterns may disrupt deeply conserved biological systems, setting the stage for contemporary cardiometabolic and neurobehavioral disorders (Figure 1).

## 2. Early Hominins: Plant-Based Diets, Fiber, and Circadian Stability

Early hominoids are widely understood to have been predominantly frugivorous, relying heavily on fruit as a primary energy source, whereas early hominins transitioned toward more diverse mixed plant-based diets that included fruits, fibrous leaves, seeds, and underground storage organs such as tubers and rhizomes [33,34]. This dietary shift is reflected in dental morphology, enamel thickness, and microwear patterns, which indicate adaptation to mechanically challenging plant materials requiring substantial mastication and prolonged oral processing [35].

Such anatomical adaptations not only reveal ecological flexibility but also imply metabolic and temporal patterns of food acquisition and consumption that were tightly integrated with environmental light–dark cycles. Plant-based, fiber-rich dietary patterns characteristic of early hominins [36] likely exerted significant influences on sleep physiology through multiple interconnected pathways [37]. High-fiber, low-glycemic foods generate gradual postprandial glucose excursions, minimizing rapid fluctuations in blood glucose levels and reducing the likelihood of nocturnal hypoglycemia. Stable glycemic profiles attenuate sympathetic nervous system activation during the night, thereby promoting sleep continuity and reducing sleep fragmentation [37,38]. Contemporary controlled feeding studies indicate that low-glycemic dietary patterns are associated with improved sleep efficiency, shorter sleep latency, and greater non-rapid eye movement (NREM) stability [37,38,39], supporting the plausibility that ancestral glycemic patterns may have favored consolidated nocturnal sleep.

In evolutionary contexts where metabolic stress and energy scarcity were common, minimizing nocturnal arousal triggered by glycemic instability would have conferred survival advantages. Beyond glycemic modulation, dietary fiber exerts systemic effects through its fermentation by colonic microbiota, leading to the production of short-chain fatty acids (SCFAs), including acetate, propionate, and butyrate. These metabolites act as signaling molecules with immunomodulatory and neuroactive properties [40,41]. Experimental evidence demonstrates that SCFAs regulate colonic regulatory T cell differentiation and anti-inflammatory immune pathways, thereby influencing systemic inflammatory tone [42]. Given the well-established relationship between inflammation and sleep architecture [12,13], reductions in pro-inflammatory signaling may enhance slow-wave sleep (SWS) depth and stability [43].

SCFAs also interact with vagal afferent pathways, providing a mechanistic route for gut-derived metabolites to influence central nervous system (CNS) function [44]. Through vagal signaling and modulation of microglial activation, SCFAs may alter neuroinflammatory states that shape sleep pressure and NREM intensity [45]. In this sense, the fiber-rich diets of early hominins likely contributed to a gut–brain milieu conducive to restorative sleep, mediated by microbiome-derived metabolites. Temporal ecology further reinforced this integration. Foraging strategies constrained feeding primarily to daylight hours, as visual acuity and predator awareness were dependent on ambient light. Such behavioral patterns would have synchronized peripheral metabolic clocks, located in liver, adipose tissue, pancreas, and gastrointestinal tissues, with central photic entrainment driven by the suprachiasmatic nucleus [46,47].

The alignment between feeding time and circadian phase optimizes insulin sensitivity, enhances mitochondrial efficiency, and reduces metabolic strain by ensuring that nutrient processing occurs during biologically appropriate windows [48,49]. Disruptions of this alignment in modern contexts are known to impair glucose tolerance and increase cardiometabolic risk, underscoring the adaptive significance of ancestral synchronization. Observational studies of contemporary hunter–gatherer populations demonstrate strong circadian entrainment to natural light–dark cycles despite moderate average sleep durations, suggesting that circadian robustness rather than absolute sleep length may have been the more critical adaptive parameter [50]. Exposure to natural light gradients, temperature fluctuations, and predictable feeding schedules appears sufficient to maintain coherent circadian phase relationships and consolidated sleep architecture.

Collectively, these lines of evidence support the view that early plant-based diets were not merely nutritional regimes but were embedded within temporally structured ecological systems that reinforced circadian stability, metabolic efficiency, microbiome diversity, and sleep integrity.

Not surprisingly, contemporary epidemiological and clinical evidence indicates that adherence to plant-based dietary patterns is associated with lower cardiometabolic burden, reduced incidence of cardiovascular disease, and decreased risk of several major cancers [51,52], likely through synergistic mechanisms involving improved insulin sensitivity, reduced systemic inflammation, enhanced endothelial function, favorable lipid modulation, and increased intake of bioactive compounds such as (poly)phenols and dietary fiber, which in turn interact with circadian metabolic pathways and gut microbial ecology [53].

## 3. Meat Incorporation: Energetics, Inflammation, and Sleep Architecture

The progressive incorporation of meat into the hominin diet, facilitated by the emergence of stone tool technologies and increasingly sophisticated butchery practices, substantially increased caloric density and nutrient bioavailability relative to predominantly plant-based foraging patterns [54,55]. Animal tissues provided concentrated sources of complete protein, heme iron, zinc, vitamin B12, and long-chain polyunsaturated fatty acids (PUFAs), including docosahexaenoic acid (DHA), which are critical for neural development and synaptic membrane integrity [55]. The energetic and micronutrient enrichment associated with meat consumption has been linked to encephalization, as expanding brain size imposed substantial metabolic demands requiring stable and energy-dense substrates [56,57,58]. A larger brain, however, also entails heightened vulnerability to metabolic and inflammatory perturbations, suggesting that shifts in dietary composition would have had cascading consequences for neurophysiology, including sleep regulation.

From a metabolic and immune perspective, red meat consumption introduces biochemical pathways with potential implications for inflammatory tone and vascular health. Dietary choline and carnitine derived from animal products can be metabolized by gut microbiota into trimethylamine (TMA), subsequently oxidized in the liver to trimethylamine-*N*-oxide (TMAO), a compound associated with atherosclerotic processes and endothelial dysfunction [59,60,61]. Although ancestral meat intake patterns differed substantially from modern industrial consumption in quantity, processing, and fat composition, the introduction of higher saturated fat loads and animal-derived metabolites likely altered systemic inflammatory dynamics. Low-grade inflammation is closely linked to sleep architecture, as pro-inflammatory cytokines such as interleukin-6 (IL-6) and tumor necrosis factor-α (TNF-α) participate in the homeostatic regulation of slow-wave sleep. Acute elevations in inflammatory mediators may enhance sleep pressure, yet chronic inflammatory states are associated with sleep fragmentation, altered rapid eye movement (REM) distribution, and diminished restorative slow-wave activity [12,13].

Contemporary evidence indicates that diets high in saturated fats correlate with reduced SWS duration and increased nocturnal arousals, suggesting that inflammatory modulation of sleep may represent a key pathway linking dietary fat composition to sleep quality [62,63]. Protein-rich animal foods also influence sleep through amino acid availability, particularly tryptophan, the precursor to serotonin and melatonin. While meat provides substantial amounts of tryptophan, its transport across the blood–brain barrier depends on competition with other large neutral amino acids, including valine, leucine, and isoleucine. The ratio of tryptophan to competing amino acids determines central serotonergic synthesis and subsequent melatonin production. Consequently, high-protein meals may have variable effects on sleep latency and architecture depending on macronutrient balance and timing. In some contexts, increased protein intake may support sleep initiation through serotonergic pathways; in others, particularly when consumed late or in large quantities, metabolic activation and thermogenesis may delay sleep onset. These nuances suggest that the incorporation of meat introduced new dimensions to the metabolic regulation of sleep, mediated by amino acid transport dynamics and neuroendocrine signaling [62,63].

Sleep–feeding interactions also operate bidirectionally. Experimental sleep restriction reliably increases appetite, particularly for energy-dense, high-fat foods [64]. In ancestral environments characterized by intermittent threat exposure, predation risk, or social vigilance, transient sleep loss may have triggered adaptive hyperphagia to replenish energy reserves. Meat, as a calorie-dense resource, would have been particularly advantageous in restoring energetic balance following periods of disrupted sleep. Thus, the integration of animal-source foods into the diet likely interacted with sleep homeostasis in a feedback loop linking arousal, energy expenditure, and compensatory intake. Overall, meat incorporation altered inflammatory load, amino acid flux, and metabolic substrate availability in ways that plausibly reshaped sleep physiology within evolving hominin ecologies [33].

The controlled use of fire further transformed dietary energetics and temporal organization. Thermal processing increased caloric extraction efficiency by gelatinizing starches, denaturing proteins, and softening connective tissues, thereby reducing mastication time and digestive effort [33,65]. Cooking decreases the metabolic costs of digestion and enhances nutrient absorption, potentially lowering postprandial thermogenesis and nocturnal metabolic activation. Reduced digestive burden during the night may have facilitated deeper, more consolidated sleep by minimizing gastrointestinal discomfort and sympathetic activation. Moreover, improved caloric efficiency would have supported larger body and brain sizes without proportionally increasing foraging time, altering daily energy allocation patterns [66]. Fire also extended social and cognitive activity into the evening hours. Low-intensity firelight modestly prolonged wakefulness, enabling storytelling, social bonding, and tool maintenance after sunset. Unlike modern artificial lighting [67], however, the spectral composition and intensity of firelight are insufficient to profoundly suppress melatonin secretion. Firelight is characterized by relatively low illuminance and a predominance of longer wavelengths in the red and orange spectrum, with substantially lower blue-light emission than contemporary electric lighting and digital screens. In contrast, modern LEDs, smartphones, tablets, and computer displays emit substantial amounts of short-wavelength blue light (approximately 450–490 nm), which strongly activates intrinsically photosensitive retinal ganglion cells containing melanopsin. These photoreceptors project directly to the suprachiasmatic nucleus and play a central role in circadian entrainment and melatonin suppression. Consequently, exposure to blue-enriched light during the evening can delay circadian phase, reduce melatonin secretion, increase alertness, and postpone sleep onset. Firelight, by comparison, produces only modest circadian effects and is generally insufficient to induce the degree of biological night disruption observed with modern electronic devices. Consequently, while evening socialization around fire may have slightly delayed circadian phase, it likely preserved endogenous melatonin rhythms and maintained overall circadian coherence [68]. This distinction helps explain why the controlled use of fire increased temporal flexibility and social complexity without producing the widespread circadian disruption that emerged much later during industrialization and intensified further in the digital era. This represents a subtle form of temporal flexibility, expanding behavioral possibilities without fundamentally disrupting the alignment between internal clocks and environmental light–dark cycles.

At the same time, cooking introduced new chemical exposures. High-temperature processing can generate compounds such as heterocyclic amines and advanced glycation end products, which are associated with oxidative stress and inflammatory responses. Chronic exposure to such compounds, even at low levels, may influence systemic inflammation and thereby subtly modulate sleep regulation over long time scales [69,70]. Nevertheless, compared with the profound circadian disruption induced by contemporary artificial light, shift work, and unhealthy dietary patterns, the evolutionary introduction of fire appears to have introduced only modest circadian modulation while substantially enhancing caloric efficiency and social complexity.

In this sense, fire and cooking represent a pivotal transition that increased energetic returns and temporal plasticity while largely preserving the fundamental alignment between diet, circadian biology, and sleep architecture.

## 4. Agriculture: High Carbohydrate Load and Sleep Regulation

The transition to agriculture marked one of the most profound dietary and ecological shifts in human evolutionary history [71]. The domestication of cereals and legumes led to increased reliance on carbohydrate-dense staple crops, substantially elevating average dietary glycemic load relative to earlier foraging patterns characterized by higher fiber content and lower glycemic variability. Refined storage, milling, and cooking techniques further enhanced starch bioavailability, accelerating glucose absorption and altering postprandial metabolic responses [33]. In parallel, dairying practices in certain pastoralist populations exerted strong selective pressures favoring lactase persistence, enabling continued lactose digestion into adulthood and providing an additional calorie-dense food source rich in protein and fat [72].

These genetic and cultural adaptations illustrate how dietary innovations reshaped both metabolic physiology and evolutionary trajectories [73]. The metabolic consequences of increased cereal consumption likely had important implications for sleep regulation. High-glycemic diets induce rapid elevations in blood glucose followed by compensatory hyperinsulinemia and subsequent glucose decline. Such fluctuations can stimulate counter-regulatory hormonal responses, including cortisol and catecholamine release, potentially increasing nocturnal arousal and sleep fragmentation. Epidemiological evidence indicates that diets characterized by high glycemic index and glycemic load are associated with increased risk of insomnia symptoms and reduced sleep quality [74].

Recurrent glycemic variability may disrupt SWS stability by enhancing sympathetic activation and altering hypothalamic–pituitary–adrenal (HPA) axis dynamics [75,76]. In individuals with limited metabolic flexibility, repeated glucose spikes could exacerbate nocturnal awakenings and impair restorative sleep architecture. Agricultural lifeways also transformed daily activity patterns and temporal organization. Sedentarization replaced mobile foraging with seasonally structured labor cycles tied to planting and harvest periods. Agricultural work often required prolonged daylight exertion during peak seasons, potentially compressing sleep duration during critical periods of food production. Conversely, winter months or post-harvest intervals may have permitted longer rest periods, introducing seasonal variability into sleep patterns [77,78].

The synchronization of human activity with agrarian calendars represents a shift from ecologically responsive foraging rhythms to more rigid, socially structured time regimes. This restructuring of labor and rest cycles may have altered circadian stability, particularly in densely populated settlements where social obligations and communal coordination increasingly shaped sleep timing. Long-term metabolic effects of carbohydrate abundance also intersect with sleep pathology. Chronic high-glycemic intake, especially when combined with reduced dietary diversity and episodic food surpluses, can promote adiposity and insulin resistance in genetically susceptible individuals [79]. Increased adiposity, particularly central fat accumulation, predisposes to obstructive sleep apnea through upper airway narrowing and reduced pharyngeal muscle tone [80]. However, obesity may not have been the sole pathway linking agricultural transitions to sleep-disordered breathing. The adoption of agriculture was accompanied by substantial changes in food texture and processing, including milling, cooking, and other techniques that reduced the mechanical demands of mastication. Evolutionary and anthropological research suggests that long-term consumption of softer foods may have contributed to reduced craniofacial development, including narrower dental arches, smaller maxillae and mandibles, and altered facial morphology [81]. Such structural changes could decrease upper airway dimensions and increase airway collapsibility during sleep, thereby predisposing individuals to obstructive sleep apnea independently of adiposity. Consequently, both metabolic consequences of carbohydrate-rich diets and biomechanical changes associated with softer agricultural foods may have acted synergistically to increase vulnerability to sleep-disordered breathing in agrarian populations.

Sleep apnea, in turn, fragments sleep architecture, reduces SWS and REM sleep, and exacerbates metabolic dysfunction through intermittent hypoxia and sympathetic activation [80].

This bidirectional relationship between metabolic dysregulation and sleep disturbance suggests that the agricultural shift toward carbohydrate-dense staples may have amplified vulnerability to sleep disorders in populations undergoing nutritional transition [82]. Furthermore, insulin resistance itself influences sleep regulation through alterations in leptin and ghrelin signaling, inflammatory cytokine production, and circadian clock gene expression [83]. Hyperinsulinemia and adipose-derived inflammatory mediators can disrupt central metabolic sensing pathways within the hypothalamus, impairing the coordination between energy status and sleep–wake control [84]. As agricultural societies expanded and population densities increased, these metabolic pressures may have interacted with infectious disease burdens and psychosocial stressors, compounding sleep disruption [85].

Collectively, the agricultural revolution introduced sustained increases in dietary glycemic load, altered temporal labor structures, and reshaped metabolic physiology. While agriculture supported demographic expansion and social complexity, it also created conditions under which glycemic variability, adiposity, and insulin resistance could impair sleep architecture. In addition, agricultural food processing practices may have influenced craniofacial morphology in ways that increased upper-airway vulnerability. In metabolically susceptible individuals, the abundance of rapidly digestible carbohydrates likely heightened sleep vulnerability, linking nutritional transition to emerging patterns of insomnia, sleep fragmentation, and sleep-disordered breathing within early agrarian populations [86].

## 5. Industrialization and Artificial Light: Circadian Disruption

The industrial era introduced environmental and behavioral conditions that profoundly disrupted the tight evolutionary coupling between light exposure, feeding patterns, metabolic regulation, and sleep [87]. The widespread availability of artificial lighting extended wakefulness well beyond sunset, fundamentally altering the photic signals that entrain the central circadian pacemaker. Exposure to artificial light, particularly short-wavelength (blue-enriched) light during the evening hours, suppresses endogenous melatonin secretion, delays circadian phase, and prolongs sleep latency [88]. Unlike the low-intensity, spectrally warm light emitted by fire, modern electric lighting and digital screens produce illumination levels sufficient to markedly attenuate nocturnal melatonin release, thereby shifting biological night later into the calendar day [89]. This phase delay reduces total sleep time in individuals constrained by fixed work or school schedules, generating chronic sleep restriction [90].

Industrial labor systems further amplified circadian disruption through shift work and round-the-clock productivity models. Rotating and night shift schedules impose wakefulness during the biological night and sleep during the biological day, producing circadian misalignment between internal clocks and external behavioral demands [91]. Such misalignment impairs glucose tolerance, elevates blood pressure, disrupts lipid metabolism, and increases long-term cardiometabolic risk [92]. At the level of sleep architecture, circadian desynchrony reduces SWS efficiency, alters REM timing, and increases sleep fragmentation. Chronic misalignment also affects hormonal rhythms, including cortisol, insulin, leptin, and ghrelin, thereby perturbing appetite regulation and reinforcing maladaptive feeding patterns [93]. The industrial reorganization of time thus represents a structural challenge to deeply conserved chronobiological systems.

Dietary transformation accompanied these temporal shifts. Industrial food systems enabled the mass production and global distribution of a wide spectrum of processed foods. Among these, dietary patterns characterized by frequent consumption of energy-dense products rich in refined carbohydrates, added sugars, certain fats, emulsifiers, and other additives have been associated with systemic low-grade inflammation oxidative stress, metabolic dysregulation, and altered gut barrier function [94]. Chronic inflammatory signaling influences sleep regulation through cytokine-mediated modulation of NREM and REM cycles [95]. While acute inflammatory responses may transiently increase sleep propensity, persistent low-grade inflammation is linked to sleep fragmentation, reduced SWS depth, and non-restorative sleep [12,13].

Additionally, highly palatable, energy-dense foods interact with dopaminergic reward pathways [96], potentially reinforcing late-night eating behaviors that further disrupt circadian alignment [97]. Industrialization has been associated with several characteristic alterations in sleep phenotypes. Average sleep duration has shortened in many industrialized societies, particularly in urban settings where artificial lighting and work demands extend into the night. Sleep fragmentation has increased due to environmental noise, psychosocial stress, and metabolic comorbidities. A population-level shift toward evening chronotype has been observed, partly driven by delayed light exposure and social schedules that encourage later bedtimes [98,99].

However, recent large-scale comparative analyses seem to challenge the simplistic “sleep restriction epidemic” narrative, with emerging cross-cultural data derived from polysomnography and actigraphy suggesting a more nuanced picture. In a synthesis of 54 population-level sleep studies and circadian analyses across industrial and non-industrial societies [100], investigators found no consistent evidence that industrialized populations experience systematically shorter sleep. On the contrary, industrial societies were characterized by the longest and most efficient sleep duration, thereby rejecting the “sleep restriction epidemic hypothesis”. At the same time, actigraphy-derived circadian function indices indicated that individuals in small-scale, non-industrial societies exhibited stronger circadian rhythmicity and greater alignment with environmental light–dark cycles, lending support to the “circadian mismatch hypothesis”. These findings imply that the primary disruption associated with industrialization may not be absolute sleep loss per se, but rather circadian desynchronization driven by artificial lighting, regulated work schedules, and social timing constraints that uncouple biological rhythms from natural zeitgebers.

On the other hand, concurrently, the prevalence of obesity and obstructive sleep apnea has risen substantially, reflecting the interaction between caloric abundance, reduced physical activity, and metabolic dysregulation [101,102]. Increased adiposity contributes to upper airway collapsibility and intermittent hypoxia, which in turn exacerbate systemic inflammation and further degrade sleep architecture. Industrialization has also reshaped the human microbiome [103]. Reduced dietary fiber intake, increased consumption of processed foods, widespread antibiotic exposure, and diminished contact with environmental microbes have collectively reduced gut microbial diversity in industrialized populations [104]. Lower microbiome diversity is associated with altered SCFA production, impaired gut barrier integrity, and increased systemic inflammation [105,106].

Given the emerging evidence linking gut-derived metabolites to vagal signaling, neuroinflammation, and sleep regulation, microbiome contraction may represent an additional pathway through which industrial lifestyles impair sleep. Changes in microbial composition may influence circadian gene expression in peripheral tissues and modulate sleep pressure through immune and metabolic signaling pathways within the gut–brain axis. In sum, industrialization introduced a convergence of circadian disruption, dietary inflammation, metabolic dysregulation, and microbiome alteration [88]. Artificial light exposure destabilized melatonin rhythms; shift work fractured temporal alignment; unhealthy foods amplified inflammatory load; and microbiome diversity declined in parallel with lifestyle modernization. These interacting forces have produced a sleep environment markedly divergent from that under which human chronobiology evolved, amplifying vulnerability to insomnia, metabolic disease, and sleep-disordered breathing. The industrial epoch thus represents a critical inflection point in the evolutionary narrative of sleep and diet, characterized by unprecedented circadian desynchronization and systemic inflammatory burden [88].

## 6. Postindustrial Globalization and Chronic Sleep Curtailment

Postindustrial globalization has intensified and accelerated many of the disruptions initiated during industrialization, producing a nutritional and temporal environment characterized by chronic sleep curtailment, psychological stress, and continuous access to highly palatable, energy-dense foods. Globalized dietary patterns are marked by high intakes of refined sugars, saturated fats, industrial seed oils, and food additives [107], often consumed in irregular temporal patterns that extend into late evening hours. These dietary characteristics interact synergistically with modern psychosocial stressors, including economic precarity, digital hyperconnectivity, and around-the-clock occupational demands [108], to generate sustained activation of stress-responsive neuroendocrine pathways.

Chronic sleep restriction has emerged as a defining feature of contemporary societies, with large-scale epidemiological analyses demonstrating that short sleep duration is prospectively associated with increased risk of obesity, cardiometabolic disease, and all-cause mortality [109,110]. The convergence of insufficient sleep and pro-inflammatory dietary exposures represents a compounding metabolic burden that diverges sharply from ancestral patterns of temporally aligned feeding and rest. Certain commercially formulated food products commonly consumed in globalized dietary patterns contain emulsifiers, stabilizers, and other synthetic additives, designed to enhance texture, shelf life, and palatability. Experimental models indicate that certain dietary emulsifiers can disrupt gut microbial ecology, impair mucosal barrier integrity, and promote low-grade intestinal inflammation [111]. Such perturbations in the gut environment may reduce microbial diversity and alter SCFA production, with downstream consequences for systemic immune tone and neuroimmune communication.

Given the bidirectional relationship between inflammation and sleep regulation, chronic dietary-induced inflammatory signaling may impair SWS depth, increase sleep fragmentation, and exacerbate subjective sleep disturbance. Furthermore, circadian misalignment, whether through irregular meal timing, late-night screen exposure, or shift work, alters peripheral clock gene expression and disrupts glucose homeostasis. When circadian desynchrony is combined with inflammatory dietary patterns, additive and potentially multiplicative impairments in insulin sensitivity, lipid metabolism, and mitochondrial function can occur, accelerating metabolic dysfunction [112].

Modern food environments also promote continuous grazing and nocturnal caloric intake, which decouple feeding rhythms from endogenous circadian phase. Late-night consumption of refined carbohydrates can provoke glycemic excursions and sympathetic activation during biological night, further delaying sleep onset and shortening total sleep time [113,114]. Repeated cycles of sleep restriction and compensatory hyperphagia reinforce weight gain, while increased adiposity predisposes to sleep-disordered breathing and further sleep fragmentation [113].

Contemporary epidemiological evidence further supports the relevance of these interactions in young adults. In a large European cohort of 962 individuals aged 18–30 years, sex-specific associations were observed between sleep behaviors, dietary habits, and somatic health indicators [115]. Late-night eating patterns and irregular sleep timing were associated with less favorable health outcomes, including higher body mass index among women and elevated systolic blood pressure among men. Frequent consumption of energy drinks was linked to lower fat-free mass index, whereas greater intake of mineral and sweetened beverages was associated with higher systolic blood pressure in men. Conversely, structured eating behaviors, particularly regular breakfast consumption and fewer daily eating episodes, were associated with higher phase angle values, a bioelectrical marker of cellular integrity and membrane function. These findings suggest that temporal dietary organization, beverage choices, and sleep behaviors interact with body composition and cardiovascular health even during early adulthood. From the perspective of evolutionary chrononutrition, such observations provide real-world evidence that modern patterns of circadian drift, late caloric intake, and stimulant beverage consumption may destabilize deeply co-evolved sleep–metabolic systems. Importantly, the results reinforce the notion that not only dietary composition, but also the timing and organization of food intake relative to sleep–wake cycles, contribute to long-term cardiometabolic resilience.

This self-perpetuating cycle illustrates how dietary globalization and chronic sleep curtailment operate within a tightly interwoven physiological network. In broader evolutionary perspective, sleep and diet represent co-evolved adaptive systems shaped by fluctuating energy availability, natural photoperiods, microbial exposure, and shifting ecological pressures [116]. Across hominin history, major dietary transitions, from plant-based foraging to increased meat consumption, thermal processing, agricultural carbohydrate reliance, industrial food refinement, and contemporary globalization, did not merely alter macronutrient composition [117].

Each transition recalibrated sleep timing, inflammatory tone, metabolic rhythms, microbiome diversity, and neuroendocrine signaling (Table 1). The modern environment, characterized by artificial light at night, persistent psychosocial stress, unhealthy food consumption, and erosion of microbial diversity, disrupts the integrated architecture that once synchronized circadian biology with ecological constraints. This evolutionary mismatch manifests in rising prevalence of cardiometabolic disease, neurodegenerative disorders, chronic inflammatory conditions, and mood disturbances [118,119].

The dissociation between endogenous circadian programs and external behavioral patterns imposes physiological strain on systems optimized for temporal coherence and nutritional variability. Re-alignment strategies grounded in “evolutionary chrononutrition” can offer a biologically coherent framework for mitigating this mismatch [120]. Aligning food intake with daylight hours, reducing consumption of unhealthy and pro-inflammatory foods, restoring microbiome diversity through fiber-rich diets and environmental exposure, practicing light hygiene to preserve nocturnal melatonin rhythms, and maintaining regular physical activity may collectively restore synchronization between metabolic and circadian rhythms [121]. Such approaches aim not to replicate ancestral environments but to re-establish fundamental temporal and metabolic principles that have governed human physiology across evolutionary time, thereby supporting sleep integrity and systemic health in post-industrial contexts.

## 7. Discussion and Future Research Directions

Future research in the emerging field of “evolutionary chrononutrition” at the interface between human nutrition, chronomedicine, and sleep science must move beyond descriptive associations toward mechanistic, longitudinal, and translational frameworks capable of disentangling causal pathways linking dietary transitions, circadian biology, microbiome ecology, and sleep architecture. A substantial body of evidence already demonstrates that sleep and metabolic regulation are bidirectionally linked, that circadian misalignment impairs glucose tolerance and lipid metabolism, that inflammatory tone shapes sleep architecture, and that dietary composition influences both microbiome diversity and systemic immune signaling. It is also well established that artificial light exposure suppresses melatonin, that shift work increases cardiometabolic risk, that dietary patterns characterized by high intakes of energy-dense foods rich in refined carbohydrates, added sugars, and multiple additives have been associated with low-grade inflammation, that microbiome diversity is reduced in industrialized populations, and that chronic short sleep predicts obesity and mortality. Experimental evidence further indicates that food additives such as emulsifiers may disrupt gut barrier integrity and promote inflammation.

Collectively, these findings provide strong support for the hypothesis that modern environments generate a convergence of circadian disruption and inflammatory dietary exposure with downstream metabolic consequences. However, several critical knowledge gaps remain. First, although epidemiological and experimental data support associations between glycemic load, inflammatory diet quality, and sleep outcomes, the precise mechanistic pathways through which specific macronutrient patterns can impair sleep microarchitecture (e.g., spectral electroencephalography (EEG) dynamics, spindle density, slow-wave slope) remain incompletely characterized.

Most human studies rely on short-term interventions or self-reported sleep measures; there is a need for longer-duration randomized controlled trials (RCTs) incorporating polysomnography, continuous glucose monitoring, inflammatory profiling, and microbiome sequencing to clarify dose–response relationships and temporal dynamics. In particular, it remains unclear whether improvements in sleep following dietary modification are mediated primarily through glycemic stabilization, inflammatory reduction, microbiome remodeling, alterations in thermogenesis, or neuroendocrine recalibration.

Second, although circadian misalignment and metabolic dysfunction are well documented, the interaction between meal timing, macronutrient composition, and chronotype has not been sufficiently disentangled. Time-restricted feeding paradigms show promise, yet optimal timing windows likely differ by chronotype, age, sex/gender, and metabolic phenotype/“metabotype”.

Moreover, the field lacks “precision chrononutrition/chronomedicine” models that integrate genetic circadian variants, peripheral clock gene expression, and individualized metabolic flexibility. Future research should incorporate multi-omics approaches, including transcriptomics of clock genes, metabolomics of substrate flux, and microbiome functional profiling, to identify subgroups most responsive to circadian realignment strategies.

Third, the role of microbiome in sleep regulation remains a frontier area. While SCFAs and inflammatory mediators have been implicated in sleep modulation, causal human evidence remains limited. Germ-free and antibiotic-treated animal models provide proof-of-concept that microbial composition influences sleep–wake cycles, yet translation to human populations requires controlled interventional trials.

Future studies should investigate whether targeted microbiome modulation, via dietary fiber diversity, fermented foods, prebiotics, probiotics, or synbiotics, can measurably alter sleep architecture, circadian phase markers, and inflammatory cytokine profiles. Importantly, research must distinguish between microbiome diversity per se and functional metabolic outputs, as compositional shifts may not fully capture bioactive metabolite dynamics.

Fourth, evolutionary mismatch frameworks are conceptually compelling but require empirical operationalization. It remains insufficiently understood which components of ancestral environments are most critical for restoring sleep–metabolic homeostasis: is the dominant driver light exposure timing, macronutrient distribution, physical activity patterns, microbial exposure, psychosocial stress reduction, or their interaction? Multifactorial intervention studies that simultaneously manipulate light hygiene, dietary composition, feeding timing, and physical activity are needed to test additive versus synergistic effects. Such designs would allow quantification of how much metabolic and sleep restoration can be achieved through realistic behavioral modification in postindustrial settings.

Fifth, interindividual variability remains underexplored. Genetic polymorphisms affecting lactase persistence, circadian clock function, inflammatory responsiveness, and appetite regulation may moderate susceptibility to sleep disruption under modern dietary exposures. Gene–environment interaction studies are required to determine whether certain ancestral adaptations (e.g., efficient carbohydrate metabolism in agrarian-descended populations) confer resilience or vulnerability under contemporary unhealthy food consumption. Similarly, sex/gender differences in sleep architecture, immune function, and metabolic regulation warrant stratified analyses, as hormonal fluctuations may interact with dietary and circadian perturbations in sex/gender-specific ways. Throughout much of human evolutionary history, females experienced unique energetic demands associated with pregnancy, lactation, and offspring care, whereas males may have faced different selective pressures related to hunting, territorial defense, and mobility. These distinct reproductive and behavioral roles likely influenced energy allocation strategies, circadian organization, and sleep architecture. For example, the energetic costs of gestation and lactation may have favored greater flexibility in sleep timing and increased sensitivity of sleep–wake regulation to metabolic signals, whereas prolonged infant care likely selected for mechanisms enabling fragmented yet resilient sleep [122,123,124]. Conversely, hunting-related activities may have favored adaptive responses to variable sleep duration and nocturnal vigilance in some male populations. Contemporary evidence demonstrates sex differences in circadian phase, sleep architecture, hormonal regulation, and metabolic responses to sleep loss [125], suggesting that at least part of this variation may reflect deep evolutionary adaptations. Understanding how evolutionary pressures shaped sex-specific sleep–diet interactions could provide important insights for precision chrononutrition and chronomedicine approaches.

Sixth, long-term neurodegenerative and psychiatric outcomes represent an area of urgent investigation. Chronic sleep fragmentation, systemic inflammation, and glycemic instability have each been independently associated with cognitive decline and mood disorders, yet longitudinal integrative studies examining their combined impact are scarce. The glymphatic clearance pathway suggests a plausible mechanism linking chronic circadian disruption to neurodegenerative risk, but it remains unclear how dietary inflammatory load or microbiome alterations modulate this process in humans. Advanced neuroimaging combined with metabolic and inflammatory biomarkers could clarify whether dietary and circadian interventions can modify trajectories of neurodegenerative vulnerability.

Finally, translational implementation science is needed. Although daylight-aligned feeding, increased consumption of healthy foods, microbiome restoration, and light hygiene are biologically coherent strategies, scalability and adherence in real-world contexts remain uncertain. Socioeconomic constraints, occupational demands, and urban infrastructure shape behavioral feasibility. Future research must integrate public health modeling, behavioral economics, and policy-level interventions to determine how chrononutrition principles can be embedded within contemporary societies without imposing unrealistic lifestyle expectations.

In summary, what is known is that sleep and diet are tightly interwoven through shared circadian, metabolic, inflammatory, and neuroendocrine pathways; that modern environments disrupt these interactions; and that such disruption is associated with cardiometabolic and neurobehavioral pathology. What remains unknown is the relative contribution of specific dietary components versus timing effects, the causal role of microbiome-mediated mechanisms in human sleep, the extent of gene–environment moderation, and the magnitude of health restoration achievable through integrated chronobiological interventions.

Addressing these gaps will require interdisciplinary collaboration spanning evolutionary biology, sleep medicine, endocrinology, microbiome science, systems biology, and public health. In parallel, substantial methodological advances are required, including the integration of artificial intelligence (AI), machine learning (ML)-based causal inference frameworks, high-dimensional multi-omics integration pipelines, wearable-derived digital phenotyping, and systems-level computational modeling to disentangle complex, nonlinear interactions across dietary, circadian, microbial, inflammatory, and neuroendocrine domains. A comprehensive, mechanistically informed research agenda has the potential not only to clarify evolutionary mismatch theory but also to inform precision strategies for restoring sleep–metabolic homeostasis in postindustrial societies (Table 2).

## 8. Conclusions

Sleep and diet represent deeply intertwined biological systems that have co-evolved under fluctuating ecological pressures, variable energy availability, natural photoperiods, and rich microbial exposures. Across major evolutionary transitions, from plant-based foraging and meat incorporation to cooking, agriculture, industrialization, and postindustrial globalization, changes in dietary composition and food processing were accompanied by parallel shifts in sleep timing, circadian alignment, inflammatory tone, metabolic flexibility, and neuroendocrine regulation.

These transitions progressively reshaped the physiological interface between energy metabolism and restorative sleep. In modern environments, artificial light exposure, unhealthy dietary patterns, irregular meal timing, psychosocial stress, and microbiome erosion collectively disrupt this integrated architecture. The resulting circadian misalignment and chronic low-grade inflammation contribute to sleep fragmentation, reduced SWS, metabolic dysregulation, and increased vulnerability to cardiovascular, cardiometabolic, neurodegenerative, and mood disorders.

This constellation of disturbances reflects an evolutionary mismatch between ancient adaptive systems and contemporary behavioral ecologies. This has profound implications in terms of global public health. Reintegrating sleep and nutrition within a chronobiological framework, emphasizing temporal alignment of feeding, dietary quality, microbiome support, light hygiene, and regular physical activity, can offer a biologically coherent strategy for restoring systemic resilience.

Within this conceptual landscape, the emerging field of “evolutionary chrononutrition” can provide a unifying theoretical and translational framework. “Evolutionary chrononutrition” integrates principles from evolutionary biology, circadian medicine, nutritional science, and microbiome ecology to examine not only what humans eat, but when and how food intake interacts with endogenous biological clocks shaped across deep evolutionary time. It posits that optimal metabolic and sleep health depend on temporal alignment between feeding rhythms, light exposure, hormonal oscillations, and peripheral clock gene expression. Rather than advocating a return to ancestral lifestyles, “evolutionary chrononutrition” seeks to restore fundamental temporal principles that governed human physiology for millennia: daylight-aligned feeding, reduction in foods characterized by high energy density, excessive added sugars, poor fiber content, and pro-inflammatory nutritional profiles, preservation of nocturnal darkness, microbiome-supportive dietary diversity, and maintenance of regular rest–activity cycles. By re-synchronizing metabolic and circadian rhythms, such strategies may enhance glycemic stability, reduce inflammatory burden, optimize sleep architecture, and improve long-term cardiometabolic and neurocognitive resilience.

Understanding sleep and diet not as isolated behaviors but as components of a unified evolutionary design reframes contemporary chronic disease not merely as a consequence of excess calories or insufficient sleep, but as a breakdown of temporal biological coherence. The maturation of “evolutionary chrononutrition” as a scientific discipline may therefore represent a critical next step in bridging evolutionary medicine with “precision chrononutrition/chronomedicine”, offering mechanistically grounded strategies to restore sleep–metabolic homeostasis in postindustrial societies.

## Figures and Tables

**Table 1:** Evolutionary Transitions in Diet and Their Integrated Impact on Sleep Physiology.

Era/Transition	Dominant Dietary Pattern	Circadian Context	Metabolic and Inflammatory Profile	Sleep Architecture Impact	Diet–Sleep Connection Mechanisms
Early Hominins (Plant-Based Foraging)	High-fiber, low-glycemic plants; fruits, leaves, tubers	Daylight-constrained feeding; strong photic entrainment	Stable glycemia; high SCFA production; low chronic inflammation	Consolidated nocturnal sleep; stable SWS	Fiber → SCFAs → immune modulation → enhanced SWS; stable glucose → reduced nocturnal arousal; feeding aligned with circadian phase
Meat Incorporation (Stone Tool Era)	Increased animal protein, saturated fats, DHA	Still light-aligned; increased energetic density	Higher caloric density; altered inflammatory load; increased amino acid flux	Variable effects: potential SWS modulation via cytokines; altered sleep latency	Tryptophan availability → serotonin/melatonin pathways; saturated fats → inflammatory cytokines affecting SWS; sleep loss → adaptive hyperphagia
Control of Fire and Cooking	Cooked starches and meats; improved digestibility	Mild extension of wakefulness via firelight; melatonin largely preserved	Reduced digestive burden; increased caloric efficiency; exposure to cooking-derived compounds	Potential deeper sleep via reduced nocturnal digestive activation; slight circadian phase delay	Lower thermogenic load → improved sleep consolidation; low-intensity light → minimal melatonin suppression
Agriculture (Neolithic Revolution)	Cereal-based, high-glycemic carbohydrates; dairy in some populations	Structured labor cycles; seasonal variation	Increased glycemic variability; rising adiposity; emerging insulin resistance	Increased insomnia risk; sleep fragmentation; early emergence of sleep apnea vulnerability	Glucose spikes → sympathetic activation; adiposity → obstructive sleep apnea; insulin resistance → circadian disruption
Industrialization	Refined carbohydrates; unhealthy foods; reduced fiber	Artificial light at night; shift work; circadian misalignment	Chronic low-grade inflammation; microbiome diversity decline	Shortened sleep duration; reduced SWS; increased fragmentation	Light → melatonin suppression; inflammation → altered NREM/REM; metabolic dysregulation → sleep instability
Postindustrial Globalization	High refined sugar, saturated fats, additives, emulsifiers	Chronic circadian disruption; 24/7 food availability	Persistent inflammatory tone; microbiome erosion; metabolic syndrome	Chronic sleep restriction; higher insomnia prevalence; increased sleep-disordered breathing	Emulsifiers → gut barrier dysfunction → inflammation; late eating → glycemic instability; sleep loss ↔ hyperphagia feedback loop

Abbreviations: DHA (Docosahexaenoic Acid); NREM (Non-Rapid Eye Movement); REM (Rapid Eye Movement), SCFA (Short-Chain Fatty Acid); SWS (Slow-Wave Sleep).

**Table 2:** Research Agenda for Evolutionary Chrononutrition.

Domain	Key Research Questions	Methodological Approaches	Primary Outcomes	Translational Implications
Temporal Feeding Alignment	How does alignment of meal timing with circadian phase influence sleep microarchitecture and metabolic flexibility?	RCTs comparing early vs. late feeding windows; polysomnography + continuous glucose monitoring; actigraphy; circadian phase assessment (DLMO)	SWS depth, REM distribution, sleep efficiency; glycemic variability; insulin sensitivity	Development of chronotype-specific meal timing guidelines
Macronutrient–Circadian Interactions	Do different macronutrient compositions (carbohydrate quality, saturated fat, protein distribution) differentially modulate clock gene expression and sleep architecture?	Controlled feeding studies; peripheral clock gene transcriptomics; metabolomics; EEG spectral analysis	Clock gene amplitude/phase shifts; inflammatory markers; spindle density; slow-wave slope	Precision dietary prescriptions for insomnia and metabolic disease
Glycemic Stability & Sleep Integrity	Does reducing glycemic variability improve sleep continuity independent of caloric restriction?	CGM-integrated dietary interventions; high vs. low glycemic index trials; HPA axis profiling	Nocturnal awakenings; cortisol rhythm amplitude; sleep latency	Chrononutritional strategies for cardiometabolic risk reduction
Microbiome–Sleep Axis	Can targeted modulation of gut microbiota restore circadian coherence and improve sleep quality?	Fiber diversity interventions; fermented food trials; shotgun metagenomics; SCFA quantification; vagal tone measurement	SCFA production; inflammatory cytokines; SWS duration; circadian robustness index	Microbiome-targeted sleep therapeutics
Artificial Light×Diet Interaction	How do evening light exposure and late caloric intake synergistically impair circadian entrainment?	Factorial experimental designs manipulating light and meal timing; melatonin profiling; wearable digital phenotyping	DLMO delay; sleep onset latency; metabolic phase misalignment	Integrated light–diet behavioral guidelines
Evolutionary Mismatch Quantification	Which ancestral temporal parameters (light exposure, fasting duration, fiber intake) most strongly predict sleep–metabolic restoration?	Multimodal intervention trials combining light hygiene, time-restricted feeding, and dietary quality; systems modeling	Circadian amplitude; inflammatory load; metabolic syndrome markers	Evidence-based evolutionary realignment protocols
Gene–Environment Interactions	Do circadian polymorphisms or metabolic genotypes modify response to chrononutrition interventions?	Genotype-stratified RCTs; GWAS-informed subgroup analysis; Mendelian randomization	Differential sleep response; metabolic adaptation rates	Precision chronomedicine frameworks
Neurodegeneration and Long-Term Outcomes	Can chrononutrition attenuate glymphatic dysfunction and neuroinflammatory pathways linked to dementia risk?	Longitudinal cohort studies; neuroimaging (glymphatic markers); inflammatory biomarker panels	Cognitive trajectories; amyloid burden; sleep fragmentation index	Preventive strategies targeting aging populations
Digital Chronobiology and AI Integration	Can AI-driven modeling integrate sleep, diet timing, glucose, microbiome, and activity data to personalize chrononutrition?	Machine learning; wearable data integration; multi-omics modeling; causal inference frameworks	Predictive accuracy for sleep and metabolic outcomes	Scalable precision chrononutrition platforms
Implementation and Public Health Translation	How can chrononutritional principles be implemented in urban, shift-working, or socioeconomically constrained populations?	Pragmatic trials; behavioral economics; policy modeling; workplace interventions	Adherence rates; sleep duration; metabolic markers; health equity metrics	Population-level chrononutrition guidelines

Abbreviations: AI (Artificial Intelligence); CGM (Continuous Glucose Monitoring); DLMO (Dim Light Melatonin Onset); EEG (Electroencephalography); GWAS (Genome-Wide Association Study); HPA Axis (Hypothalamic–Pituitary–Adrenal Axis); RCT (Randomized Controlled Trials); SCFAs (Short-Chain Fatty Acid); SWS (Slow-Wave Sleep).

## Data Availability

No new data were created.
